# The impact of organizational commitment on turnover intention of substitute teachers in public primary schools: Taking psychological capital as a mediator

**DOI:** 10.3389/fpsyg.2022.1008142

**Published:** 2022-09-23

**Authors:** Kexuan Zhu, Xinyi Wang, Man Jiang

**Affiliations:** Dhurakij Pundit University, Bangkok, Thailand

**Keywords:** substitute teachers, organizational commitment, turnover intention, psychological capital, public primary schools

## Abstract

This research aimed to explore the impact of organizational commitment on turnover intention of substitute teachers in public primary schools in Xuzhou, and applied psychological capital as a mediator variable to establish a research model. A questionnaire was conducted with 400 substitute teachers using convenience sampling. The results show that organizational commitment has a negative yet significant effect on turnover intention. It also shows positive impact on psychological capital. Furthermore, psychological capital is shown to negatively impact turnover intention, while having a mediating effect between organizational commitment and turnover intention.

## Introduction

The COVID-19 pandemic has blurred the lines between personal work and personal life, resulting in a more unequal work-life balance for teachers (Schmidt-Crawford et al., 2021), and more pressure on teachers (Crook, 2020). On the other hand, statistics and facts have shown that substitute teachers are under great pressure (Vorell, 2011; Gershenson, 2012), and their vocational satisfaction is lower than that of non-substitute teachers (Topchyan and Woehler, 2021), which will potentially cause substitute teachers to have higher turnover intention (Kurniawaty et al., 2019). Teacher Mobility has a serious impact on the quality of school teaching, on students’ academic developments, and even on the school community (Räsänen et al., 2020). Thus, the key factors and influencing mechanisms that affect the turnover intention of substitute teachers must be explored.

One of the basic doctrines of social exchange theory is that relationships develop over time into trusting, loyal, and mutual commitments (Cropanzano and Mitchell, 2005). Social exchange theory claims that employees are connected by a network made of ties whose strength influences their intention to keep or leave their jobs (Holtom et al., 2008). Researchers believe that studying turnover intentions may benefit an organization more than actual turnover (Islam et al., 2016). There are various factors that may influence turnover intention and contribute to job-hopping behavior, including organizational support, job satisfaction, commitment, burnout, emotional exhaustion, job stress, career plateau, person-organizational fit, work engagement and job embeddedness (Lee et al., 2018; Huang et al., 2021; Kim and Kim, 2021). These reasons are often the motivation for employees to voluntarily change their workplace (Lake et al., 2018). Research has indicated that organizational commitment affects turnover intention (Wasti, 2003; Jehanzeb et al., 2013; Guzeller and Celiker, 2019), and this impact in the current research would be extremely amplified. However, results of empirical studies are scarce. Therefore, investigating this topic further to obtain additional evidence to remedy deficiencies in past research is worthwhile.

In addition to organizational commitment, one known way to effectively influence employees’ willingness to leave is to increase their stress tolerance (Çelik, 2018). Psychological capital consists of four dimensions: optimism, hope, self-efficacy and flexibility (Luthans et al., 2007a), which all affect the attitude of employees conducting their work and improving their endurance (Avey et al., 2009). As an individual psychological variable that can be managed, developed and improved, psychological capital can promote the decrease of turnover intention (Imran et al., 2017; Koo et al., 2020). Moreover, psychological capital often mediates turnover intention studies (Jeon et al., 2016; Bouzari and Karatepe, 2017; Sepeng et al., 2020). Therefore, we believe that psychological capital also plays a mediation role in the relationship between organizational commitment and turnover intention.

There are two remarkable innovations in this research: first, although there is some research on the turnover intention of school teachers (Alev and Bozbayindir, 2021; Liu et al., 2021), there is no distinction between substitute teachers and in-service teachers in these studies, and research on turnover intention of substitute teachers is lacking. Therefore, we considered substitute teachers as the sample in the present study to compensate for the shortcomings of past studies. Second, the current research applies organizational commitment, psychological capital, and turnover intention to the theory of social exchange to understand the mediating role of psychological capital in the relationship between organizational commitment and turnover intention. However, the influence mechanism of this relationship is still unclear. Therefore, the current study uses public primary school substitute teachers as the sample, organizational commitment as the independent variable, psychological capital as the intermediary variable, and turnover intention as the dependent variable to explore the three aspects of the organizational commitment, psychological capital and turnover intention. The inter-influence mechanism provides a new direction for reducing substitute teachers’ turnover intention and further enriches social exchange theory.

The research questions in the current study are as follows:

1. Does the organizational commitment of substitute teachers in public primary schools affect their turnover intention?
2. Does the organizational commitment of substitute teachers in public primary schools affect their psychological capital?
3. Does the psychological capital of substitute teachers in public primary schools affect their turnover intention?
4. Does the psychological capital of substitute teachers in public primary schools play a mediating role in the influence of organizational commitment on turnover intention?

## Literature review

### Theoretical basis-social exchange theory

Based on social exchange theory, we seek to understand the influence mechanism among organizational commitment, psychological capital, and turnover intention. Social exchange theory is the study of human behavior at the micro level using theories from sociology, psychology and economics (Homans, 1958), making it one of the most powerful tools for understanding workplace behavior (Malik et al., 2011). Social exchange theory deals with three principles including rationality, reciprocity and specificity principle to explain the relationships between employee and employer (Foa and Foa, 2012). The first rationality principle reasons that employees will have association with that organization which can provide desirable rewards and satisfy its employees needs and wants. The second reciprocity principle theorize that social relationship is always reciprocal between employee and employer. The third specificity principle postulates that only reciprocity type can endure an exchange relationship between the employees and an organization (Foa and Foa, 2012).

The establishment of a relationship between the organization and employees results in the employees exchanging their loyalty to the organization and their own labor for the rewards given by said organization (Rhoades and Eisenberger, 2002). Conversely, if the individual feels that the relationship is not good for them, they are likely to abandon the relationship (Lai et al., 2014). Substitute teachers are paid less and perform better than non-substitute teachers, raising questions of distributive justice and potentially encouraging counterproductive behavior (Chernyak-Hai and Rabenu, 2018).

In gist, the three principles of social exchange theory underpinned the reciprocal relationships between organizational commitment, psychological capital and turnover intention. According to social exchange theory, the more loyal an individual is to the organization, the higher his psychological capital and the lower his turnover intention.

### Organizational commitment and turnover intention

Becker (1960) was the first to define organizational commitment where he argued that as ‘Unilateral efforts’ to an organization increased, the tendency to embrace all kinds of work within the organization was called organizational commitment. Some scholars believe that organizational commitment is an internalized code of conduct (O’Reilly and Chatman, 1986). This integration of internal pressures aligns the employee’s behavior with the thoughts and interests of the organization (Wiener, 1982). Similarly, organizational commitment can also be viewed as a person’s psychological attitude toward organizational relationships, including whether the individual is willing to stay in the organization (Allen and Meyer, 1990). Meyer and Allen (1991) divided organizational commitment into three dimensions, namely, emotional commitment, continuing commitment and normative commitment. It can be seen from this that organizational commitment is a link between individuals and organizations (Mathieu and Zajac, 1990). Organizational commitment is interpreted as a function of workplace attitudes, behaviors, and management (Islam et al., 2018). In the current study, we adopt the definition of Allen and Meyer (1990) and define organizational commitment as a person’s psychological attitude toward organizational relationships, including whether the individual is willing to remain in the organization.

Today, long-term employee retention and reduced employee turnover have become huge challenges for every organization (Top and Ali, 2021). Ideal turnover is for incompetent employees, when talented, skilled, and capable employees leave against the employer’s wishes (Shim, 2010). Porter et al. (1974) suggests that the avoidance behavior that employees exhibit when they are dissatisfied with their jobs is called turnover intention. Turnover is divided into voluntary turnover and involuntary turnover (Shepherd et al., 2020). Numerous studies have demonstrated a positive relationship between personal intentions and departure behavior. Individuals with high overturn intentions tend to be perceived as less productive and always try to find a way out of the organization (Hamza et al., 2021). Therefore, academic scholars should rely on employee turnover intention as an alternative to actual turnover to reduce organizational losses (Anwar and Qadir, 2017).

As a variable of employees’ attitudes toward the organization, organizational commitment is a core predictor of turnover behavior, exit tendency and organizational citizenship behavior (Mathieu and Zajac, 1990; Sinclair et al., 2005). Organizational commitment can help organizations retain a talented workforce (Islam et al., 2014). Consequently, we proposed hypothesis 1 of this study.

*H1*: Substitute teachers’ organizational commitment significantly and negatively affects their turnover intention.

### Organizational commitment and psychological capital

Luthans et al. (2005) first put forward the theory of psychological capital and believe that psychological capital refers to a person’s growth and development of a positive psychological state. At the same time, psychological capital emphasizes the psychological origin of an individual, which includes four aspects: self-efficacy, hope, optimism, and resiliency (Gooty et al., 2009). Self-efficacy is a perception or belief about an individual’s own abilities, whereas optimism is a positive expectation and therefore has no relation to an individual’s actual abilities (Luthans et al., 2010). The hope is that one sticks to the goal, adjusts the channel actively and flexibly, and achieves your goal at some point; when encountering difficulties, being able to persevere, recover quickly, and catch up to achieve success is resilience (Luthans et al., 2007b). As a shared latent ability, psychological capital is believed to be critical to human motivation, cognitive processing, effort success, and performance in the workplace (Peterson et al., 2011). In the current study, we adopted the definition of Luthans et al. (2007b) and defined psychological capital as a person’s growth and development of a positive psychological state.

The more employees perceive their organization’s support of them, the more commitment they will give to the organization (Aube et al., 2007), and in this harmonious organizational environment, the employee’s psychological capital will also increase (Hui et al., 2014). In previous studies is has been shown that organizational commitment is positively related to psychological capital (Idris and Manganaro, 2017; Nguyen and Ngo, 2020), which also shows that these two variables are closely related. Psychological capital has been shown to have a positive and significant effect on organizational commitment (Youssef and Luthans, 2007; Yildiz, 2018), but the effect of organizational commitment on psychological capital has not been confirmed, which is also a gap in the current research. Consequently, we proposed hypothesis 2 of this study.

*H2*: Substitute teachers’ organizational commitment significantly and positively affects their psychological capital.

### Psychological capital and turnover intention

Psychological capital is a psychological resource that enables employees to adapt to challenging environments, maintain a positive work attitude in the work environment, and remain in their organization (Luthans et al., 2005; Kim et al., 2017). If a person’s psychological capital is higher, turnover intention will be lower (Avey et al., 2008). Therefore, psychological capital has a significant negative impact on turnover intention (Karatepe and Karadas, 2014; Karatepe and Avci, 2017; Yan et al., 2021). Based on the above discussion, we propose Hypothesis 3 of this study.

*H3*: Substitute teachers’ psychological capital significantly and negatively affects their turnover intention.

### The mediation role of psychological capital in the relationship between organizational commitment and turnover intention

To fully understand how organizational commitment influences substitute teachers’ turnover intention, we should also consider the possible mediating role of employee characteristics, especially related to individuals’ psychological assets. Luthans et al. (2005) argue that psychological capital is a positive psychological resource that can be influenced by various organizational or leadership variables. As an individual psychological variable that can be managed, developed, and improved, psychological capital can promote the decrease of turnover intention (Imran et al., 2017; Koo et al., 2020). Moreover, psychological capital often plays a mediation role in turnover intention studies. For example, Jeon et al. (2016) found that psychological capital acts as a mediator in the relationship between transformational leadership and turnover intention. Bouzari and Karatepe (2017) research found that servant leadership style affects employees’ turnover intention through the mediating effect of employees’ psychological capital. Sepeng et al. (2020) found that psychological capital mediates the relationship between authentic leadership and turnover intention. Therefore, we proposed hypothesis 4 of this study.

*H4*: Psychological capital has a mediating effect between substitute teachers’ organizational commitment and turnover intention.

## Materials and methods

### Research framework

**Figure 1:**
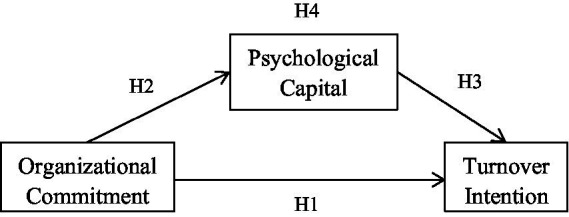
Research framework.

A research framework was constructed according to the aforementioned hypotheses (Figure 1).

### Research instruments

The organizational commitment, turnover intention, psychological capital scales employed by this study are detailed as follows:

#### Organizational commitment scale

The organizational commitment scale was devised by Allen and Meyer (1996) with satisfactory reliability. The scale contains three dimensions and 18 items. The scale contains 3 dimensions and 18 items. The affective commitment has six items including “I have a strong sense of belonging to my school,” and the normative commitment has six items including “I should be fully committed to school,” and six questions such as “I will lose a lot if I leave my current school” for sustained commitment. The scale adopts a 5-point scale (1 = strongly disagree; 5 = strongly agree). Cronbach’s α was 0.733 for affective commitment, 0.705 for sustained commitment and 0.869 for normative commitment, respectively.

#### Turnover intention scale

The turnover intention scale was devised by Farh et al. (1998). The scale consists of one dimension and 4 items, an example of which is “I’ve always wanted to quit my current job.” The scale adopts a 5-point scale (1 = strongly disagree; 5 = strongly agree). The third question is a reverse coded item. The scale has Cronbach’s α of 0.84 and homogeneity reliability of 0.78, so the scale is more reliable.

#### Psychological capital scale

The psychological capital scale in this study uses the revised item and structure of the psychological capital of Luthans et al. (2007a) questionnaire by Wen et al. (2009). The scale contains four dimensions and 16 items and adopts a 5-point scale (1 = strongly disagree; 5 = strongly agree). An example of the self-confidence dimension is “I believe I can contribute to discussions of school organizational strategies”; for the hope dimension, such as “I can think of many ways to achieve my current work goals”; the resilience dimension includes “I am able to handle a lot of things at once in my work”; “I always see the bright side of things in my work” is for the optimistic dimension. The Cronbach’s α of each scale and total scale is between 0.7031 and 0.8125.

### Research participants

The questionnaire was distributed to substitute teachers in 18 urban public primary schools in Xuzhou, Jiangsu Province, covering the five municipal districts.

### Procedure

Quantitative methods are gaining popularity as an efficient modeling tool (Rad et al., 2022a), so this study used the questionnaire method. The study was divided into pilot test and formal test. Two questionnaires were administered to different participants.

The pilot test questionnaire was conducted among 110 substitute teachers in public primary schools in Xuzhou using the convenience sampling method. There were 101 valid questionnaires, the response rate was 91.82%. The online questionnaire was distributed online by Wechat from February 12, 2022 to March 3, 2022. The pilot test results show that the extreme value *t*-test of each item is >0.3, the correlation between the corrected item and the total score is >0.4, and the factor loading is >0.5; in the reliability analysis, the Cronbach’s α is >0.8; in confirmatory factor analysis, the CR value of each questionnaire is >0.7, and the AVE value is >0.4. According to the results, no inappropriate questions were found, so a total of 38 questions were reserved to continue the formal questionnaire survey.

In the formal questionnaire survey, partcipants submitted the survery through WeChat group. With reference to Tinsley and Tinsley (1987), the number of questionnaires distributed should be combined with the number of questions, and the ratio of the number of items to the sample size should be between 1:5 or 1:10. There are 38 items in this survey, and the maximum ratio is 1:10. So this study needs at least 380 valid samples. Considering that some questionnaires may be invalid, 400 questionnaires are given out. 384 valid questionnaires were collected.

### Statistical analysis

Firstly, descriptive statistics and Pearson’s correlation coefficients were performed for the organizational commitment, turnover intention and psychological capital variables using SPSS 22.0. Finally, we explored the specific relationship between pairs of the three variables and examined the mediation role of psychological capital in the impact of organizational commitment on turnover intention.

## Results

The following is an analysis of the descriptive statistics of the participants, variable descriptive statistics and correlation analysis, the predictive power of organizational commitment to turnover intention, and the mediating effect of psychological capital on the relationship between organizational commitment and turnover intention.

### Descriptive statistics of the participants

A total of 400 questionnaires were distributed and returned; 384 valid outcomes result in a valid return rate of 96%, constituted by 171 (44.53%) male respondents and 213 (55.47%) female respondents. In terms of age distribution, the number of respondents in the group aged 20–25 is the same as that of the group aged 25–30: 124 (32.29%). The age groups of 30–35 and 35–40 consist of 65 (16.93%) and 49 (12.76%) respondents respectively, while 22 (5.73%) respondents were aged 40 or above. For educational attainment, 71 respondents (18.49%) graduated with an associate degree, 251 people (65.36%) had undergraduate degrees only, and 62 respondents (16.15%) had master’s degrees or above.

### Variable descriptive statistics and correlation analysis

As shown in Table 1, the mean (*M*) and standard deviation (*SD*) of each variable are as follows: organizational commitment (*M* = 2.149 and *SD* = 0.640), turnover intention (*M* = 3.640 and *SD* = 1.018), and psychological capital (*M* = 2.945 and *SD* = 1.063). Because all three scales were rated on 5-point scales, organizational commitment and organizational commitment exhibited moderate-to-low average scores, and turnover intention exhibited moderate-to-high average scores. Organizational commitment was negatively related to turnover intention (*r* = −0.660, *p* < 0.001), organizational commitment was positively correlated with psychological capital (*r* = 0.570, *p* < 0.001), psychological capital was negatively correlated with turnover intention (*r* = −0.686, *p* < 0.001). The correlation coefficient of each pair was 0.570–0.686, indicating no collinearity.

**Table 1:** Variable descriptive statistics and correlation analysis.

Variable	M	SD	OC	TI	PC
OC	2.149	0.640	1		
TI	3.640	1.018	−0.660^***^	1	
PC	2.945	1.063	0.570^***^	−0.686^***^	1

^***^p < 0.001.^**^p < 0.01.^*^p < 0.05.

OC, organizational commitment; TI, turnover intention; PC, psychological capital.

### Regression analysis

This study examined the mediating effect of substitute teachers’ psychological capital on the relationship between organizational commitment and turnover intention based on the premise that the effects of demographic variables, that is, gender, age, and education are controlled. As shown in Table 2, the organizational commitment of substitute teachers significantly and negatively impacted turnover intention (*β* = −0.652, *t* = −16.653, *p* < 0.001) in Model 1, and therefore, hypothesis H1 checks out. The organizational commitment of substitute teachers significantly and positively affected psychological capital (*β* = 0.556, *t* = 13.103, *p* < 0.001) in Model 2; therefore, hypothesis H2 is valid. In Model 3, after adding the mediating variable psychological capital, substitute teachers’ organizational commitment had a significant negative effect on turnover intention (*β* = −0.387, *t* = −9.546, *p* < 0.001) and their psychological capital significantly and negatively influenced turnover intention (*β* = −0.476, *t* = −11.675, *p* < 0.001), and hence, hypothesis H3 is confirmed. The β value for the effect of the organizational commitment of substitute teachers on turnover intention decreased from −0.652, up to the significant level, in Model 1 to −0.387, up to the significant level, in Model 3. Thus, psychological capital plays a partial mediation role in the effect of organizational commitment on turnover intention in substitute teachers, and so, hypothesis H4 is valid. Furthermore, this study used the Sobel test to verify the mediating effect and used unstandardized regression coefficients and standard errors to calculate it. The formula for the Sobel test which is *z*-value = a × b/SQRT(b^2^ × s_a_^2^ + a^2^× s_b_^2^), a = raw (unstandardized) regression coefficient for the association between the organizational commitment and the psychological capital·s_a_ = standard error of a·b = raw coefficient for the association between the psychological capital and the turnover intention (when the organizational commitment is also a predictor of the turnover intention)·s_b_ = standard error of b. *Z* value of >1.96 represents a significant mediating effect (Sobel, 1982), and the present study showed that psychological capital (*Z* = −8.698, *p* < 0.001) has a significant mediating effect. In Model 3, the VIF was <10 and no covariance problem was noted.

**Table 2:** Mediating effect of psychological capital on the relationship between organizational commitment and turnover intention.

Variable	Model 1		Model 2		Model 3		VIF
TI		PC		TI	
	*β*	*t*	*β*	*t*	*β*	*t*	
Male	0.059	1.518	−0.028	−0.651	0.046	1.377	1.025
20–25	0.094	1.139	0.021	0.233	0.104	1.469	4.614
26–30	0.084	1.014	−0.089	−0.994	0.041	0.582	4.632
31–35	0.047	0.660	−0.091	−1.182	0.003	0.057	3.411
36–40	0.073	1.111	−0.079	−1.106	0.035	0.628	2.895
College	0.071	1.379	0.070	1.249	0.104	2.357^*^	1.800
University	0.043	0.846	0.012	0.219	0.049	1.119	1.780
OC	−0.652	−16.653^***^	0.556	13.103^***^	−0.387	−9.546^***^	1.506
PC					−0.476	−11.675^***^	1.527
*R* ^2^	0.444		0.345		0.592		
Adj *R*^2^	0.432		0.331		0.582		
*F*	37.389^***^		24.711^***^		60.373^***^		

^***^p < 0.001; ^**^p < 0.01; ^*^ *p* < 0.05.

β is the standardized regression coefficient. OC, organizational commitment; TI, turnover intention; PC, psychological capital.

Based on the above analysis, psychological capital has a significant partial mediating effect between organizational commitment and turnover intention. Details are given in Table 2.

## Discussion

Firstly, the results of this study show that the organizational commitment of substitute teachers in public primary schools in Xuzhou has a significant negative effect on turnover intention. This is consistent with the findings of Wasti (2003), Jehanzeb et al. (2013), and Guzeller and Celiker (2019). Substitute teachers have precarious jobs and low organizational commitment to the school, and they may lose their jobs due to changes in policies and positions. Instead of being resigned, they actively look for a better platform, so their tendency to leave is high.

Second, this study found that organizational commitment positively affects the psychological capital of substitute teachers. Although some previous literature has demonstrated a positive correlation between organizational commitment and psychological capital (Idris and Manganaro, 2017; Nguyen and Ngo, 2020), this is the first study to demonstrate that organizational commitment positively predicts psychological capital. Therefore, improving the organizational commitment of substitute teachers will also help improve their psychological capital.

Third, the results of this study show that the psychological capital of substitute teachers in Xuzhou public primary schools has a significant negative impact on turnover intention. This is consistent with the study of Avey et al. (2008), Karatepe and Karadas (2014), Karatepe and Avci (2017), and Yan et al. (2021). In the current educational environment, substitute teachers face pressures from various aspects, such as low wages, instability, and psychological imbalance. Maintaining a good level of psychological capital is not only particularly important to reduce the turnover intention of substitute teachers, but also for the improvement of education quality and the educational environment.

Finally, the results show that the psychological capital of substitute teachers in public primary schools in Xuzhou has a partial mediating effect between organizational commitment and turnover intention. When substitute teachers receive high organizational commitment, they will be more motivated to move towards their goals, have more confidence in choosing plans to achieve their goals, and be better able to demonstrate that they can achieve their goals through the necessary effort, even in the face of challenging work. The confidence brought by successful completion will also make positive attributions and enhance their willpower to recover when the encounter problems and difficulties, which can stimulate the psychological capital of substitute teachers, and under the effect of this positive psychology, they will perform the job itself and the school is more likely to generate higher satisfaction, which in turn reduces substitutes’ turnover intention.

## Practical implications

It is worth considering increasing the use of the psychological capital questionnaire in the process of selecting substitute teachers to improve the stability of those hired as substitute teachers. The reliability and validity of the questionnaire of psychological capital is in accordance with standards of psychometrics, meaning it could be used as a measurement tool. Understanding and mastering the psychological capital status of substitute teachers in the initial stage of employment is important for carrying out targeted intervention and stabilizing them. It is better to grasp the change of the substitute teachers’ psychological capital in real time as far as possible, and find the regular contents to provide the empirical basis for making more effective intervention programs.

Organizational support and learning environments are among the aspects that provide HR with the highest level of return, especially in terms of its organizational commitment (Islam et al., 2013). In order to reduce the turnover tendency of substitute teachers, education departments and schools should strengthen their organizational commitment from the following aspects. First, attach importance to the cultivation and guidance of the substitute teachers’ professional quality, and provide them with more opportunities to learn and practice. Both formal and informal training can have a positive impact on teachers’ knowledge, skills and competencies (Rad et al., 2022b). And employee’s training affects positively organizational commitment and it ultimately reduces turnover and turnover intentions (Bodjrenou et al., 2019). Second, conduct educational exchanges and experience-sharing activities for new and old teachers, and provide favorable conditions for substitute teachers to have the opportunity to participate in targeted school-based and out-of-school training and to improve their professional qualities and educational and teaching capabilities, help them grow from novice teachers as quickly as possible. Knowledge sharing in an organization promotes organizational commitment among employees, because it encourages active social interaction to achieve mutual benefits (Curado and Vieira, 2019). Third, substitute teachers who have excellent performance should be given material and spiritual rewards to create a good office environment. Employees care about the support and benefits the organization provides them (Nazir et al., 2018). The school should actively help these substitute teachers solve certain difficulties in their lives and ensure the teachers feel the warmth of the organization directly to enhance their commitment to the organization, so as to improve psychological capital and reduce turnover intention. Finally, in the first few months of working, the work experience of substitute teachers is crucial to the formation of emotional commitment. Shanks et al. (2020) found that organizational onboarding support for new teachers can promote new teacher retention. The school should give more attention to first-time and novice teachers in life and work. For example, holding relevant seminars or having a variety of social or league-building activities for new teachers.

## Research limitations and future directions

The main limitation of this study is that the coverage of primary schools is not wide enough as only substitute teachers from 18 public primary schools in the city of Xuzhou were selected. More data would allow for extrapolation of the results to public primary schools outside of the city of Xuzhou. Moreover, mixed research methods including interviews and the survey would be better for in-depth investigation. Last, a survey administered to permanent teachers would be beneficial for comparing substitute teachers and non-substitute teachers.

## Data availability statement

The raw data supporting the conclusions of this article will be made available by the authors, without undue reservation.

## Ethics statement

The studies involving human participants were reviewed and approved by Dhurakij Pundit University. The patients/participants provided their written informed consent to participate in this study. Written informed consent was obtained from the individual(s) for the publication of any potentially identifiable images or data included in this article.

## Author contributions

KZ designed the study, analyzed the data. XW drafted the manuscript. MJ assisted in analyzing and interpreting the data. All authors contributed to the article and approved the submitted version.

## Conflict of interest

The authors declare that the research was conducted in the absence of any commercial or financial relationships that could be construed as a potential conflict of interest.

## Publisher’s note

All claims expressed in this article are solely those of the authors and do not necessarily represent those of their affiliated organizations, or those of the publisher, the editors and the reviewers. Any product that may be evaluated in this article, or claim that may be made by its manufacturer, is not guaranteed or endorsed by the publisher.
